# ACE-domain selectivity extends beyond direct interacting residues at the active site

**DOI:** 10.1042/BCJ20200060

**Published:** 2020-04-09

**Authors:** Gyles E. Cozier, Lizelle Lubbe, Edward D. Sturrock, K. Ravi Acharya

**Affiliations:** 1 Department of Biology and Biochemistry, University of Bath, Claverton Down, Bath BA2 7AY, U.K.; 2 Department of Integrative Biomedical Sciences, Institute of Infectious Disease and Molecular Medicine, University of Cape Town, Observatory, 7925 Cape Town, Republic of South Africa

**Keywords:** enzyme inhibition kinetics, metalloproteases, protein crystallography, site-directed mutagenesis

## Abstract

Angiotensin-converting enzyme (ACE) is best known for its formation of the vasopressor angiotensin II that controls blood pressure but is also involved in other physiological functions through the hydrolysis of a variety of peptide substrates. The enzyme contains two catalytic domains (nACE and cACE) that have different affinities for ACE substrates and inhibitors. We investigated whether nACE inhibitor backbones contain a unique property which allows them to take advantage of the hinging of nACE. Kinetic analysis showed that mutation of unique nACE residues, in both the S2 pocket and around the prime subsites (S′) to their C-domain counterparts, each resulted in a decrease in the affinity of nACE specific inhibitors (SG6, 33RE and ketoACE-13) but it required the combined S2_S′ mutant to abrogate nACE-selectivity. However, this was not observed with the non-domain-selective inhibitors enalaprilat and omapatrilat. High-resolution structures were determined for the minimally glycosylated nACE with the combined S2_S′ mutations in complex with the ACE inhibitors 33RE (1.8 Å), omapatrilat (1.8 Å) and SG6 (1.7 Å). These confirmed that the affinities of the nACE-selective SG6, 33RE and ketoACE-13 are not only affected by direct interactions with the immediate environment of the binding site, but also by more distal residues. This study provides evidence for a more general mechanism of ACE inhibition involving synergistic effects of not only the S2, S1′ and S2′ subsites, but also residues involved in the sub-domain interface that effect the unique ways in which the two domains stabilize active site loops to favour inhibitor binding.

## Introduction

Angiotensin-converting enzyme (ACE, EC 3.4.15.1) is a zinc metalloprotease which has traditionally been known for its role in blood pressure regulation via the renin–angiotensin–aldosterone system (RAAS). During the last 20 years, there has been an increasing interest in the function of ACE in other biological systems. The somatic form of ACE (sACE) is expressed on the surface of endothelial, epithelial, neuroepithelial and immune cells [1] as a type I transmembrane protein. Although formed from a single polypeptide chain, this protein has two catalytically active extracellular domains, the N- and C-domain (nACE and cACE, respectively), which are separated by a short linker region. The high degree of homology between the sACE domains (60% overall sequence similarity and 89% active site identity) [2] suggests that they originated by an evolutionary gene duplication event and were conserved due to differences in their physiological function. sACE cleaves a remarkable range of substrates through both endo- and exopeptidase action. These include angiotensin I, enkephalins, kinins, neurotensin, formyl-Met-Leu-Phe, substance P [3], gonadotropin-releasing hormone (GnRH), also known as luteinizing hormone–releasing hormone (LH-RH) [4], N-acetyl-Ser-Asp-Lys-Pro (AcSDKP) [5] as well as the amyloid-beta-peptide (Aβ) [6,7]. cACE is primarily responsible for blood pressure regulation via angiotensin I cleavage [8,9] whereas the peptides GnRH, AcSDKP and Aβ are preferentially cleaved by nACE [5–7,10,11], and bradykinin is hydrolyzed by both domains [11].

Apart from decreasing blood pressure, the clinical use of non-domain-selective ACE inhibitors has been associated with various antifibrotic effects. Since this is largely attributed to the accumulation of the ubiquitously expressed AcSDKP, selective N-domain inhibition holds the potential for the treatment of fibrosis without affecting blood pressure [12,13].

ACE has also been implicated in the immune response and recently overexpression of ACE in macrophages and neutrophils was shown to cause an increase in oxidative metabolism and cellular ATP; however, the peptide substrate is currently unknown [14,15]. Further highlighting the importance of controlled domain-specific ACE inhibition this link to the immune response is due to cACE activity [16].

**Figure 1.**
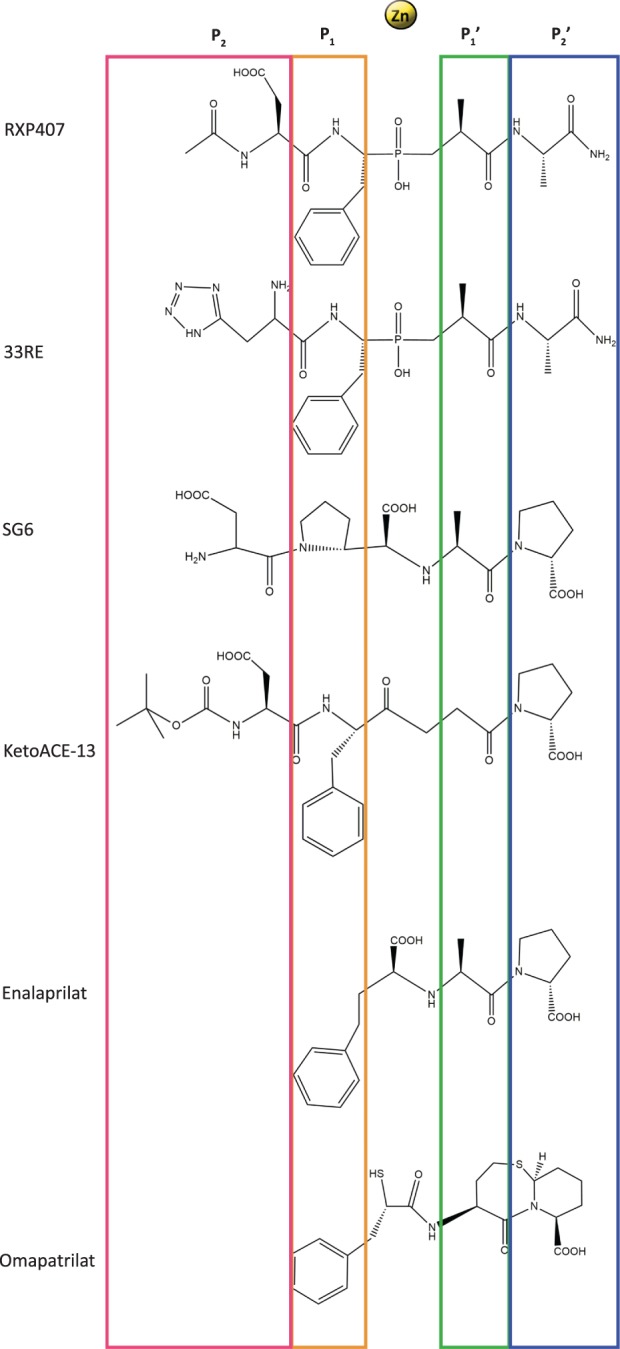
Chemical structures of the ACE inhibitors studied here. Inhibitor moieties are annotated with Schechter–Berger nomenclature based on their interactions with the non-prime and prime subsites flanking the active site Zn^2+^.

At present, there are only a handful of ACE inhibitors with substantial N-selectivity, of which the phosphinic peptidomimetic compounds RXP407 [17] and its analogue 33RE [18] show the most promise. Peptidic inhibitors are, however, generally considered poor drug candidates because of their low oral bioavailability, poor solubility and susceptibility to degradation by proteases [19]. Functional groups essential for N-selectivity, therefore, need to be identified to enable the design of clinically suitable nACE inhibitors. RXP407 has N-terminal acetylated aspartate (P_2_), l-pseudophenylalanine (P_1_), l-pseudoalanine (P_1_′) and C-terminal amidated alanine (P_2_′) moieties that, respectively, bind to the S_2_, S_1_, S_1_′ and S_2_′ subsites of the ACE active site (Figure 1). Its fragment-based design analogue 33RE is identical in structure apart from the P_2_ moiety being replaced by an aminomethyl tetrazole. Mutagenesis studies showed that the three orders of magnitude N-selectivity of these two compounds largely depends on interactions with unique nACE residues of the S_2_ subsite [18,20]. However, unique prime subsite residues distal to the inhibitor binding site were also required to fully account for the selectivity of these compounds. Each ellipsoid domain of ACE is divided into two sub-domains flanking the active site cleft. Previous normal-mode analysis and isothermal titration calorimetry studies have suggested that ligand binding is mediated by hinging and subsequent opening or closing of the active site cleft [21,22]. It was recently shown via molecular dynamics simulations that the mechanism of hinging differs between wild-type nACE and a mutant where eight unique active site residues were replaced by their cACE counterparts [23]. Differential scanning fluorimetry further showed that these residues were crucial for the thermal stabilization of the protein upon 33RE binding. These nACE residues from the S_2_ subsite (Y369 and R381), the S_1_′ subsite (T358) and distal prime region (S260, E262, D354, S357 and E431), referred to as S_2_ (YR) and S′ (S_1_′ and distal prime), synergistically control the selectivity of 33RE and their mutation to cACE counterparts (F391, E403, V380, T282, S284, E376, V379 and D453, respectively) completely abolished selectivity. The only unique direct interaction was between the P_2_ tetrazole of 33RE and Y369. The unique distal prime residues indirectly facilitated 33RE binding by favouring the protein's hinging towards active site cleft closure. In nACE, these residues formed an attractive polar sub-domain interface whereas in cACE, this interface was hydrophobic and caused repulsion between the sub-domains.

Previous attempts at designing more drug-like ACE inhibitors with this mechanism of action have had limited success. Although the addition of an aspartate moiety in the P_2_ position of a relatively non-selective ACE inhibitor backbone such as ketoACE to produce ketoACE-13 [24] or enalaprilat to produce SG6 (also known as compound 16) [25] increased the level of N-selectivity, it was still far inferior to that of 33RE. It is important to understand the reason behind this to ensure future success in the design of highly N-selective inhibitors. We, therefore, investigated whether the 33RE backbone contains a unique property which allows it to take advantage of this hinging behaviour of nACE by determining the mechanisms of action for a range of ACE inhibitors differing in their backbones and degrees of N-selectivity, namely: omapatrilat, enalaprilat, SG6 and ketoACE-13 (Figure 1). This included kinetic studies using the S_2_, S′ and combined S_2__S′ mutated nACE (S_2_-nACE, S′-nACE and S_2__S′-nACE, respectively), as well as solving high-resolution crystal structures of minimally glycosylated nACE with the combined S_2__S′ mutations (N389-S_2__S′-nACE) in complex with SG6, 33RE and omapatrilat.

## Materials and methods

### Enzymes for kinetic characterization

Truncated wild-type or mutant proteins used for kinetic characterization were fully N*-*glycosylated. Wild-type N-domain (nACE), C-domain (cACE) and the S_2_-, S′- and S_2__S′-nACE mutants were previously created and cloned into pcDNA3.1+ (Invitrogen) for mammalian cell expression [23]. Chinese hamster ovary K1 (CHO-K1) cells stably overexpressing these ACE proteins were cultured and the proteins purified using lisinopril–sepharose affinity chromatography, as previously described [26].

### Ligand preparation

The fluorogenic substrate Abz-FRK(Dnp)P-OH was a kind gift from Prof A.K. Carmona, Universidade Federal de São Paulo. The peptide was dissolved in 100% DMSO and the concentration determined by measuring the absorbance at 365 nm and extinction coefficient of 17 300 M^−1 ^cm^−1^. Subsequent dilutions from the 1 mM stock of were made in assay buffer (50 mM HEPES, pH 6.8, 10 μM ZnCl_2_, 200 mM NaCl). The inhibitors 33RE [18], SG6 [25] and ketoACE-13 [24] were synthesized previously whereas enalaprilat dihydrate and omapatrilat were obtained from Sigma–Aldrich. Lyophilized 33RE, SG6 and enalaprilat were dissolved in distilled water to yield stock concentrations of 10 mM. KetoACE-13 was dissolved in 100% methanol to a stock concentration of 50 mM, followed by dilution in deionized distilled water to 10 mM. Since the dimerization of omapatrilat has been previously described [27], fresh 10 mM stocks of inhibitor were prepared in DMSO directly prior to kinetic analysis. The integrity of omapatrilat at ambient temperature was monitored at 50 µM by HPLC (Agilent 1260 Infinity HPLC System with a Poroshell 120 EC-C 18 column) over the timeframe of each assay, as previously described [27]. A 20 µl injection volume was used with a 0.5 ml/min flow rate with A: 1% ACN, 0.1% TFA; B: 95% ACN, 0.1% TFA; and 0–100% B over 10 min; 214 nm.

Subsequent dilutions of all inhibitors were prepared from stock solutions in an assay buffer consisting of 50 mM HEPES pH 6.8, 200 mM NaCl and 10 μM ZnCl_2_

### Kinetic characterization of substrate hydrolysis

Binding and hydrolysis of the fluorogenic Abz-FRK(Dnp)P-OH substrate was determined as previously described in an assay buffer consisting of 50 mM HEPES pH 6.8, 200 mM NaCl and 10 µM ZnCl_2_ [28]. Briefly, a 50 μM solution of Abz-FRK(Dnp)P-OH was prepared in assay buffer (50 mM HEPES, pH 6.8, 10 μM ZnCl_2_, 200 mM NaCl) and variable volumes added to a 96-well plate on ice. A constant volume of the enzyme was added to all wells and the final volume adjusted to 300 μl with buffer to give a constant amount of enzyme with a range of substrate concentrations. The hydrolysis of Abz-FRK(Dnp)P-OH was continuously monitored at excitation and emission wavelengths of 320 nm and 420 nm, respectively, at ambient temperature using a fluorescence spectrophotometer (Cary Eclipse, Varian Inc.). The amount of active protein was determined immediately after purification by calculating the specific activity. Final enzyme concentrations in these assays were 10 nM for nACE and S′-nACE, and 5 nM for S_2_-nACE, S_2__S′-nACE and cACE. Kinetic constants were calculated by analysis of the initial reaction velocities (at <10% substrate hydrolysis) with the Michaelis–Menten model in GraphPad Prism v.6.0.

### Kinetic characterization of inhibitor binding

To evaluate inhibitor binding affinity, a modified Abz-FRK(Dnp)P-OH assay was used. Serial dilutions of an appropriate range were prepared for each inhibitor in the aforementioned assay buffer. An appropriate concentration of enzyme was similarly prepared and incubated with an equal volume of inhibitor for 15 min at 22°C after which 20 µl volumes of the reactions were aliquoted to a 96-well plate. The volume per well was increased to 300 µl by the addition of Abz-FRK(Dnp)P-OH on ice at an appropriate concentration to allow detectable inhibition. The final substrate and enzyme concentrations for these assays were as follows: For SG6 and omapatrilat: 4 µM Abz-FRK(Dnp)P-OH with 2.6 nM enzyme; for enalaprilat: 4 µM Abz-FRK(Dnp)P-OH with enzyme at 1.3 nM (nACE or cACE), 0.66 nM (S′-nACE or S_2_-nACE) or 0.33 nM (S_2__S′-nACE); for ketoACE-13: 2.6 nM nACE with 4 µM Abz-FRK(Dnp)P-OH, 0.7 nM S′-nACE with 3 µM Abz-FRK(Dnp)P-OH, 0.2 nM S_2_-nACE with 3 µM Abz-FRK(Dnp)P-OH, 0.2 nM S_2__S′-nACE with 2 µM Abz-FRK(Dnp)P-OH or 0.5 nM cACE with 3 µM Abz-FRK(Dnp)P-OH. Control (zero inhibitor) reactions containing the appropriate concentration of methanol or DMSO (in the case of ketoACE-13 or omapatrilat, respectively) were included to correct for any possible vehicle-induced effects on the reaction rate.

Initial reaction velocities were analyzed in GraphPad Prism v.6.0 using the Morrison equation $\left(Y=(1-((((E_{t}+X+(K_{i}^{\mathrm{app}}))-(((E_{t}+X+(K_{i}^{\mathrm{app}}){)}^{2})-4*E_{t}*X{)}^{0.5}))/(2*E_{t}))\right)$, where *Y* is *V*_i_/*V*_o_, *X* is the final inhibitor concentration and *E*_t_ is the final enzyme concentration) to obtain $K_{i}^{\mathrm{app}}$ values. Under conditions of classical inhibition where competition with substrate affected $K_{i}^{\mathrm{app}}$ (which only applied to the ketoACE-13 kinetics), the Cheng–Prusoff equation ($K_{i}\;=\;K_{i}^{\mathrm{app}}/(1+[\text{S}]/K_{m\;})$; where [S] is the final substrate concentration) was used to obtain *K*_i_ values [29].

### Engineering of a minimally glycosylated active site mutant

nACE is routinely crystallized in a minimally glycosylated form where six of the nine N-linked glycosylation sites have been mutated to glutamine residues. The resulting construct, N389-nACE, only retained glycosylation at sites 3 (Asn45), 8 (Asn416) and 9 (Asn480) [30]. To enable crystallization of the S_2__S′-nACE active site mutant, the asparagine residues at sites 1 (Asn9), 2 (Asn25), 4 (Asn82), 5 (Asn117), 6 (Asn131) and 7 (Asn289) were converted to glutamine residues. Sites 1, 2, 4, 5 and 6 were situated upstream of the active site mutations and could, therefore, be removed by molecular cloning. The N3789-S_2__S′-nACE construct was generated by performing sequential *Bsu*36I and *Eco*RI (Thermofisher Scientific^TM^) restriction enzyme digests on the fully glycosylated pcDNA3.1+ S_2__S′-nACE and the minimally glycosylated pcDNA 3.1+ N3-nACE [31] and ligating the N-terminal region of the latter to the C-terminal fraction of the former, containing the active site mutations. A double digest of *Eco*RI and *Xba*I was performed to screen for the presence of a ∼2000 bp coding region and thus the generation of the pcDNA 3.1+ N3789-S_2__S′-nACE construct. Glycosylation site 7 (Asn289) was located amongst the active site mutations and as such was mutated to glutamine via site-directed mutagenesis using the Kapa HiFi PCR kit (Roche Molecular Systems Inc.) and previously designed primers [30]. Positive introduction of the desired mutation, and thus the generation of the pcDNA 3.1+ N389-S_2__S′-nACE construct, was confirmed by restriction enzyme screening for the simultaneously introduced *Nde*I site. Additionally, bidirectional nucleotide sequencing was performed to confirm the presence of all active site mutations and N > Q mutation of sites 1, 2, 4, 5, 6 and 7.

### Heterologous expression of minimally glycosylated mutant ACE

The construct was transfected into Chinese hamster ovary (CHO-K1) cells using the calcium phosphate Profection® Mammalian Transfection System (Promega Corp.), heterologously expressed and the protein purified from culture medium via lisinopril–sepharose affinity chromatography, as previously described [26]. Protein purity was assessed by SDS–PAGE and Coomassie staining. Purified protein was quantified by absorbance with the use of a NanoDrop® spectrophotometer and extinction coefficient of 162 070 M^−1 ^cm^−1^ for nACE.

### X-ray crystallographic studies

N389-S_2__S′-nACE was pre-incubated with the ligands for 1 h at room temperature using a 4 : 1 v/v ratio of 5 mg ml^−1^ protein (in 50 mM HEPES, pH 7.5, 0.1 mM PMSF) and inhibitor (20 mM 33RE or SG6, 5 mM omapatrilat). Hanging drops of 1 µl of the protein-inhibitor complex mixed with an equal volume of reservoir solution were set up. The standard condition for nACE (30% PEG 550 MME/PEG 20000, 0.1 M Tris/Bicine pH 8.5, and 60 mM divalent cations, Molecular Dimensions Morpheus A9) was used to test for N389-S_2__S′-nACE crystallisation, and it produced crystals of the same morphology.

X-ray diffraction data were collected on stations i03 (33RE) and i04-1 (omapatrilat and SG6) at the Diamond Light Source (Didcot, U.K.), with the crystals kept at a constant temperature (100 K) using a nitrogen stream. Images were collected using PILATUS3 6 M (33RE) and PILATUS-6M-F (omapatrilat and SG6) detectors (Dectris, Switzerland). Raw data images were indexed and integrated with DIALS [32], and then scaled using AIMLESS [33] from the CCP4 suite [34]. Initial phases were obtained by molecular replacement with PHASER [35] using N389-nACE PDB code 6F9V [36] as the search models. Further refinement was initially carried out using REFMAC5 [37] and then Phenix [38], with COOT [39] used for rounds of manual model building. Ligand and water molecules were added based on electron density in the mFo-DFc Fourier difference map. MolProbity [40] was used to help validate the structures. Crystallographic data statistics are summarized in Table 3. All figures showing the crystal structures were generated using CCP4mg [41], and schematic binding interactions are displayed using Ligplot+ [42].

## Results

It is known that synergism between unique S_2_ subsite and sub-domain interface residues is important for the N-selectivity of 33RE [23]. To evaluate whether this mechanism of selectivity is applicable to other ACE inhibitor backbones site-directed mutagenesis, enzyme kinetics and X-ray crystallography were employed.

### Kinetic characterization of substrate hydrolysis

The truncated (single domain) wild-type N-domain (nACE), wild-type C-domain (cACE) and N-domain mutant constructs S_2_-nACE (Y369F and R381E), S′-nACE (S260T, E262S, D354E, S357V, T358V and E431D) and S_2__S′-nACE (S260T, E262S, D354E, S357V, T358V, Y369F, R381E and E431D) were heterologously expressed in CHO-K1 cells [23]. The secreted proteins were purified to homogeneity via lisinopril-coupled sepharose affinity chromatography and SDS–PAGE showed single bands for each construct corresponding to ∼90 kDa for nACE/mutants and 70 kDa for cACE (data not shown). Hydrolysis of the quenched fluorogenic substrate Abz-FRK(Dnp)P-OH was characterized and it was observed that the S_2_-nACE and S_2__S′-nACE displayed lower binding affinities (*K*_m_ of 11.92 μM and 12.40 μM, respectively) and higher catalytic turnover rates (*k*_cat_ of 4.5 s^−1^ and 6.0 s^−1^, respectively) compared with both wild-type domains and S′-nACE (Table 1). The overall efficiency of substrate hydrolysis (*k*_cat_*/K*_m_) was, however, comparable between all proteins studied in agreement with our previous study [23].

**Table 1.** Catalytic efficiency of Abz-FRK(Dnp)P-OH hydrolysis by wild-type and mutant proteins

Construct	*K*_m_ (μM)	*k*_cat_ (s^−1^)	*k*_cat_/*K*_m_ (s^−1^ μM^−1^)
nACE	7.54 ± 1.7	1.9 ± 0.3	0.26 ± 0.04
S′-nACE	4.51 ± 2.0	1.2 ± 0.5	0.27 ± 0.03
S_2_-nACE	11.92 ± 1.0	4.5 ± 1.4	0.38 ± 0.14
S_2__S′-nACE	12.40 ± 1.6	6.0 ± 0.7	0.48 ± 0.03
cACE	6.30 ± 1.1	2.6 ± 0.5	0.42 ± 0.05

Initial reaction velocities (<10% of substrate hydrolysis) were analyzed using the Michaelis Menten model in GraphPad Prism^®^ v6.01 for determination of *K*_m_ and *k*_cat_ values (represented as mean ± SD of three independent experiments).

### Kinetic characterization of inhibitor binding

**Figure 2:**
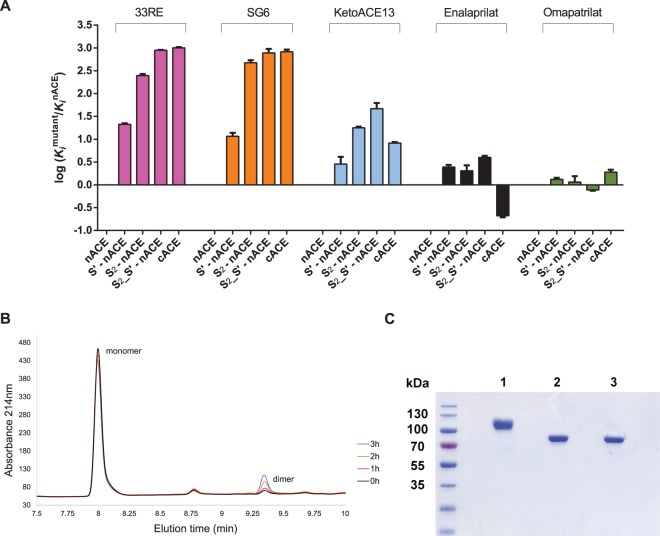
. Effect of mutation on N-selective inhibition, integrity of omapatrilat at ambient temperature as monitored by HPLC and SDS–PAGE gel illustrating the decreased molecular mass of the active site mutant N389-S_2__S′-nACE. (A) Inhibitor binding affinities (*K*_i_ values) for wild-type cACE or nACE mutants are shown on a log scale relative to nACE with error bars representing mean ± SD of at least two independent experiments. (B) Omapatrilat (50 μM in assay buffer) was analyzed using a Poroshell 120 EC-C 18 column on the Agilent 1260 infinity HPLC system. Twenty microliters of the sample was injected onto the column immediately after preparation or following incubation at ambient temperature for 1, 2 or 3 h. Conditions: A: 1% ACN, 0.1% TFA; B: 95% ACN, 0.1% TFA; 0–100% B over 10 min; 0.5 ml/min, 214 nm. Peaks corresponding to the monomer and dimer were observed at 7.99 and 9.34 min, respectively. (C) SDS–PAGE of purified nACE proteins (1) fully glycosylated S_2__S′-nACE, (2) minimally glycosylated N389-nACE and (3) minimally glycosylated N389-S_2__S′-nACE.

Previous kinetic studies reported that 33RE was a high-affinity inhibitor of nACE (*K*_i_ of 11.21 nM) and had ∼1000-fold selectivity over cACE [18] with S′-nACE, S_2_-nACE and S_2__S′-nACE mutations, respectively, leading to decreases in binding affinity of 22-, 254- and 900-fold [23] (Table 2). This shows that the S_2_ mutations have a greater effect than the S′ on 33RE affinity of nACE and that upon combining all eight mutations in S_2__S′-nACE, a synergistic effect occurs so that the affinity for 33RE is similar to that seen for cACE (Figure 2A). Here, we studied the effect of these mutations on the binding of two reportedly moderately N-selective inhibitors, SG6 and ketoACE-13, and two non-selective inhibitors, omapatrilat and enalaprilat.

**Table 2.** Affinity of inhibitor binding to the wild-type and mutant proteins

Inhibitors	Proteins	*K*_i_ (nM)	Selectivity ($K_{{}_{i}}^{{}^{\mathrm{cACE}}}/K_{i}^{\mathrm{nACEormutant}}$)
33RE*	nACE	11.21 ± 0.735	1006
	S′-nACE	237.22 ± 13.60	48
	S_2_-nACE	2794 ± 156	4
	S_2__S′-nACE	10 009 ± 157	1.1
	cACE	11 278 ± 410	1
SG6	nACE	0.093 ± 0.02	825
	S′-nACE	1.09 ± 0.1	70
	S_2_-nACE	44.05 ± 2.8	1.7
	S_2__S′-nACE	72.93 ± 7.5	1.1
	cACE	76.77 ± 4.3	1
KetoACE13	nACE	1000 ± 270	8
	S′-nACE	2990 ± 1000	3
	S_2_-nACE	17 810 ± 1100	0.5
	S_2__S′-nACE	47 750 ± 13 600	0.2
	cACE	8250 ± 430	1
Enalaprilat	nACE	0.75 ± 0.20	0.2
	S′-nACE	1.85 ± 0.21	0.09
	S_2_-nACE	1.56 ± 0.41	0.1
	S_2__S′-nACE	3.00 ± 0.24	0.05
	cACE	0.16 ± 0.01	1
Omapatrilat	nACE	0.37 ± 0.05	2
	S′-nACE	0.49 ± 0.04	1
	S_2_-nACE	0.43 ± 0.13	2
	S_2__S′-nACE	0.29 ± 0.01	2
	cACE	0.70 ± 0.09	1

Inhibitor binding constants (*K*_i_ values) were determined from inhibition assays via Morrison plots and are represented as mean ± SD of at least two independent experiments. For SG6, enalaprilat and omapatrilat $K_{i}^{\mathrm{app}}$ was equal to *K*_i_ due to the tight-binding nature of inhibition, whereas *K*_i_ values for ketoACE13 were calculated from $K_{i}^{\mathrm{app}}$ values using the Cheng–Prusoff equation [29].

* Lubbe et al. [23].

To enable comparison between inhibitors of different potencies, Morrison plots were constructed from initial reaction velocities in the presence of different inhibitor concentrations (*V*_i_) as a fraction of velocity in the absence of inhibitor (*V*_0_) for the wild-type and mutant enzymes. Omapatrilat, enalaprilat and SG6 showed picomolar to nanomolar potency for all enzymes tested and exhibited a tight-binding mode of inhibition (Table 2). Since no competition with the substrate was detected for these inhibitors it was assumed that $K_{i}^{\mathrm{app}}$ approximates *K*_i_, as recently described for omapatrilat [27]. KetoACE-13 displayed significantly lower potency (micromolar range) compared with the other inhibitors tested and a classical mode of inhibition. In this case, $K_{i}^{\mathrm{app}}$ no longer approximated *K*_i_ and the latter were calculated using the Cheng–Prusoff equation where $K_{i}\;=K_{i}^{\mathrm{app}}/(1+([\text{S}]/K_{m}))$ [29]_._

The diprolyl analogue of enalaprilat, SG6, displayed *K*_i_ values of 0.093 nM and 76.77 nM for nACE and cACE, respectively, and thus 825-fold N-selectivity similar to that of 33RE. The effect of mutation follows a similar trend to that of 33RE with S_2_ and sub-domain interface residues synergistically controlling N-selectivity. The S′ mutations resulted in a modest 11-fold decrease in affinity (*K*_i_ of 1.09 nM), followed by a more pronounced decrease in 474-fold with S_2_-nACE (*K*_i_ of 44.05 nM) and a drastic decrease in 784-fold when all mutations were combined in S_2__S′-nACE (*K*_i_ of 72.93 nM), thereby yielding cACE-like affinity. From these data, it can be concluded that for 33RE and SG6 some, or all, of the S′ residues play a significant role in their binding, although this appears to be mainly if the affinity from the direct binding interactions (S_2_ residues) has also been reduced.

The binding affinities obtained here for ketoACE-13 (*K*_i_ values of 1000 nM and 8250 nM for nACE and cACE, respectively (Table 2)) were higher and the degree of N-selectivity slightly lower than those reported previously by Sharma et al. [24]. This discrepancy is partly due to the use of initial inhibitor concentrations as opposed to final inhibitor concentrations in *K*_i_ calculations by the previous authors, which meant a 4-fold increase in their values. Interestingly, the 8-fold N-selectivity of ketoACE-13 was markedly lower compared with that of 33RE and SG6 determined here under identical conditions, despite the presence of a P_2_ aspartate for interaction with the unique Y369 and R381 residues of the S_2_ subsite. Nevertheless, the mutations studied here elicited the same trend of decreased binding affinity for ketoACE-13 as for SG6 and 33RE with synergism existing between the S_2_ and S′ residues (Figure 2A). This was rather unexpected given the low degree of N-selectivity observed for this compound. The S′ mutations led to a 3-fold reduction in binding affinity (*K*_i_ of 2990 nM) which, considering the low selectivity of ketoACE-13, was a drastic change towards cACE-like inhibition. Interestingly, S_2_-nACE and S_2__S′-nACE had even lower affinities than cACE for ketoACE-13 with *K*_i_ values of 17 810 nM and 47 750 nM, respectively. Since the active site of S_2_-nACE is essentially equivalent to that of cACE, some property distal to the cACE active site appears to minimize the effect of Y369 and R381 loss on its inhibition by ketoACE-13. This property is likely lacking in nACE and it, therefore, depends on the S_2_ and sub-domain interface residues for inhibition by ketoACE-13.

This property potentially also affects the binding of enalaprilat. This compound binds with a 5-fold greater affinity to cACE than nACE (*K*_i_ values of 0.75 nM and 0.16 nM for nACE and cACE, respectively), yet mutation ofS′, S_2_ and S_2__S′ counterintuitively resulted in slightly decreased binding affinities (2.5-, 2- and 4-fold, respectively) compared with nACE. This suggests that these residues facilitate nACE inhibition even in the absence of a P_2_ moiety and that cACE relies on some factor distal to the binding site for its effective inhibition. Addition of a P_2_ aspartate, as in SG6, likely strengthens the intrinsic synergism between nACE subsites to bring about selectivity.

Omapatrilat displayed sub-nanomolar potency for nACE and cACE with *K*_i_ values of 0.37 nM and 0.70 nM, respectively, in line with previous reports [27]. Omapatrilat was evaluated via HPLC during the assay timeframe to monitor potential dimer formation. A small fraction of dimer (2.56%) was detected immediately after solubilization and this increased by a mere 0.86% after 1 h at ambient temperature (Figure 2B), confirming that the monomer was the dominant species during kinetic characterization. Even after 3 h of incubation at ambient temperature, only 11% dimer was detected. This was lower than that reported previously [27], suggesting that the current buffer conditions (50 mM HEPES pH 6.8) are more suitable for future studies with omapatrilat. Interestingly, omapatrilat binding was not affected by any of the mutations tested (Table 2), suggesting that the effects observed for enalaprilat were overruled by attributes like the strong zinc-chelating sulfhydryl and carbonyl moieties or more favoured orientation within the active site of the former.

### Engineering of a minimally glycosylated S_2__S′-nACE

ACE is heavily glycosylated and contains 10 N-domain and seven C-domain potential N-linked glycosylation sites of which all except one site per domain are occupied by complex-type glycans. To enable crystallization, minimally glycosylated versions of the truncated wild-type domains were previously engineered by Asn > Gln mutation at certain positions [30,43]. The nACE mutant N389-nACE, which only retained glycosylation at sites 3 (Asn45), 8 (Asn416) and 9 (Asn480), was catalytically active and the most amenable to crystallization. At present, more than 20 crystal structures have been solved of N389-nACE complexed to a variety of ligands. Here, we created a minimally glycosylated version of S_2__S′-nACE (N389-S_2__S′-nACE) to gain structural insight into the pronounced effects that these mutations have on inhibitor binding. The N-linked glycosylation sites 1 (Asn9), 2 (Asn25), 4 (Asn82), 5 (Asn117) and 6 (Asn131) were converted to glutamine residues by subcloning the N-terminus of S_2__S′-nACE with that of N389-nACE. Replacement of site 7 (Asn289), however, required the use of site-directed mutagenesis due to its location between the E262S and D354E mutations of the active site. The minimally glycosylated active site mutant N389-S_2__S′-nACE was successfully created, stably transfected, expressed and purified as illustrated by the lower molecular mass on SDS–PAGE compared with S_2__S′-nACE (Figure 2C).

### Crystallisation of N389-S_2__S′-nACE inhibitor complexes

The minimally glycosylated N389-nACE readily crystallises in the presence of ligands using a single condition that was identified from initial screens, and no screening around this condition is needed even for new ligand complexes. Therefore, this condition (Morpheus A9 from Molecular Dimensions: 30% PEG 550 MME/PEG 20000, 0.1 M Tris/Bicine pH 8.5 and 60 mM divalent cations) was tried for N389-S_2__S′-nACE as both the native enzyme, and in complex with 33RE, SG6 and omapatrilat. We hypothesized that structures with these inhibitors would provide the most information about the S_2__S′ mutations since the kinetic data showed that omapatrilat is entirely independent and 33RE/SG6 greatly dependent on these residues for binding affinity. Even after multiple attempts, and using streak seeding from other N389-nACE crystals, no native (without the presence of a ligand) N389-S_2__S′-nACE crystals grew. The N389-nACE protein also rarely crystallises in the native form, and even when crystals do form, they always have elements from the purification and crystallization conditions bound in the active site.

However, N389-S_2__S′-nACE readily crystallised in the presence of the three ligands (33RE, SG6 and omapatrilat). All the complexes were in the *P*1 space group with two molecules of the protein in the asymmetric unit. The structures were determined at high resolutions of 1.8 Å for both 33RE and omapatrilat, and 1.7 Å for SG6 (Table 3).

**Table 3.** X-ray data collection and refinement statistics

	33RE	SG6	Omapatrilat
Resolution (Å)	[74.14–9.86] (1.83–1.80)	[75.11–9.31] (1.73–1.70)	[64.01–9.86] (1.83–1.80)
Space group	P1	P1	P1
Cell dimensions (*a*,*b*,*c*) angles (*α*,*β*,*γ*)	73.2, 77.1, 82.9 Å 88.9, 64.5, 75.2°	73.6, 78.2, 83.8 Å 88.6, 64.4, 74.6°	74.4, 78.8, 89.7 Å 92.4, 106.0, 114.6°
Molecules/asymmetric unit	2	2	2
Total/unique reflections	980 600/141 976	1 188 881/172 853	1 093 096/157 824
Completeness (%)	[99.3] 97.4 (96.2)	[99.4] 97.2 (95.4)	[99.3] 97.2 (96.1)
*R*_merge_	[0.041] 0.083 (0.923)	[0.021] 0.084 (0.836)	[0.017] 0.069 (0.692)
*R*_pim_	[0.016] 0.034 (0.371)	[0.008] 0.034 (0.372)	[0.007] 0.028 (0.291)
<*I*/*σ*(*I*)>	[42.7] 11.5 (1.9)	[65.9] 11.6 (1.6)	[66.8] 13.9 (2.0)
CC_1/2_	[0.999] 0.999 (0.652)	[1.000] 0.999 (0.511)	[1.000] 0.999 (0.776)
Multiplicity	[7.1] 6.9 (7.1)	[7.8] 6.9 (6.0)	[7.8] 6.9 (6.5)
Refinement statistics			
*R*_work_/*R*_free_	0.200/0.237	0.188/0.221	0.182/0.222
*R*_msd_ in bond lengths (Å)	0.010	0.009	0.010
*R*_msd_ in bond angles (°)	1.076	0.967	1.011
Ramachandran statistics (%)			
Favoured	97.0	97.9	98.2
Allowed	2.8	2.1	1.7
Outliers	0.2	0.0	0.1
Average B-factors (Å^2^)			
Protein	38.62	34.50	36.02
Ligand	46.29	48.06	55.15
Water	37.64	37.86	39.05
Number of atoms			
Protein	9836	9963	10 088
Ligand	337	375	395
Water	795	888	989
PDB code	**6TT1**	**6TT3**	**6TT4**

Inner shell, overall and outer shell statistics are given in square brackets, un-bracketed and round brackets, respectively.

### Overall structure of the N389-S_2__S′-nACE mutant

**Figure 3.**
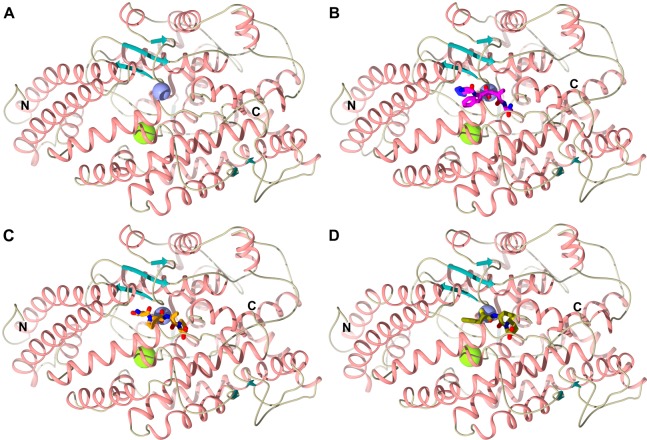
Schematic representation of the overall structures of N389-nACE and N389-S_2__S′-nACE/inhibitor complexes. (**A**) Wild-type N389-nACE, (**B**) N389-S_2__S′-nACE/33RE complex, (**C**) N389-S_2__S′-nACE/SG6 complex and (**D**) N389-S_2__S′-nACE/omapatrilat complex. 33RE, SG6 and omapatrilat inhibitors are shown as magenta, orange and olive sticks, respectively. Zinc and chloride ions are depicted as lilac and green spheres, respectively, with helices, β-strands and loops coloured in rose, dark cyan and tan, respectively.

The overall fold of N389-nACE, with the molecular replacement model PDB 6F9V being a typical example, has previously been shown to be a mainly α-helical ellipsoid comprising of two sub-domains (Figure 3A). These sub-domains form a closed structure containing a two-lobed cavity (the S′ and S subsites) with the active site zinc at the junction of the two lobes. Contained within the first 100 residues is a flexible region that has been described as ‘lid-like’, and has been suggested to control peptide access into the active site [43]. This typically shows higher temperature factors (B-factors) than the rest of the structure and is usually poorly defined in one molecule of the asymmetric unit as a result of the crystal packing. The degree of flexibility shown does not appear to be caused by the ligand bound as it can vary from crystal to crystal of the same complex.

All three of the N389-S_2__S′-nACE complex structures (with 33RE, SG6 and omapatrilat inhibitors) show a very similar overall fold to each other and are also essentially the same as seen for N389-nACE (Figure 3B–D). This is reflected in the RMSD values, for 602 Cα atoms observed in all aligned structures, which vary from 0.34 to 0.52 Å (Table 4).

**Table 4.** Comparison of the overall structures of N389-S_2__S′-nACE inhibitor complexes and N389-nACE

		N389-S_2__S′-nACE		
		33RE	Omapatrilat	SG6
N389-nACE		0.43	0.48	0.48
N389-S_2__S′-nACE	33RE	—	0.47	0.34
	Omapatrilat	—	—	0.52

RMSD values (Å) for 602 Cα atoms observed in all structures. Values were generated using the ‘Structure Alignment and Superposition with Gesamt‘ program on CCP4 cloud.

**Figure 4.**
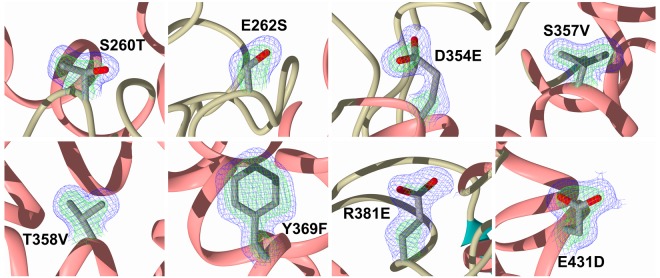
N389-S_2__S′-nACE structures confirm the presence of the mutations. The final 2mFo-DFc (blue, contoured at 1σ level) and the omit mFo-DFc (green, contoured at 3σ level) electron density maps for the N389-S_2__S′-nACE/SG6 structure clearly show the eight mutations, which are depicted as sticks with α-Helices, β-strands and loops shown in rose, dark cyan and tan, respectively.

**Figure 5.**
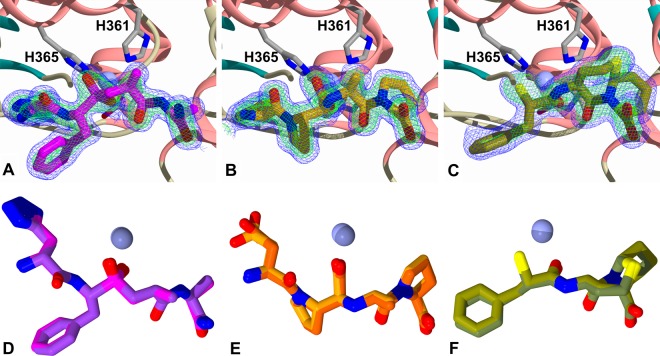
Inhibitors bound to N389-S_2__S′-nACE. (**A**) 33RE, (**B**) SG6 and (**C**) Omapatrilat shown as schematic representations bound to N389-S_2__S′-nACE with the final 2mFo-DFc (blue, contoured at 1σ level) and the omit mFo-DFc (green, contoured at 3σ level) electron density maps. Zinc ions are depicted as lilac spheres with the coordinating side chains shown as sticks (Glu389 is obscured in this view, so not labelled). α-Helices and β-strands are shown in rose and dark cyan, respectively. Overlays are shown of (**D**) 33RE, (**E**) SG6 and (**F**) Omapatrilat bound to wild-type N389-nACE and N389-S_2__S′-nACE. The inhibitors from wild-type structures are coloured dark magenta for 33RE (PDB 4BXK), orange red for SG6 (PDB 6EN5) and dark olive for omapatrilat (PDB 6H5X), with the zinc ions as dark lilac spheres. N389-S_2__S′-nACE bound inhibitors are coloured magenta, orange and olive for 33RE, SG6 and omapatrilat, respectively, with lilac sphere zinc ions.

The high resolution of the structures presented here, in particular for the SG6 complex, confirm the presence of the S_2__S′ mutations that had been introduced (Figure 4). In all three N389-S_2__S′-nACE complex structures (33RE, SG6 and omapatrilat) clear, unambiguous electron density for the bound ligands was observed in the mFo-DFc omit maps (Figure 5A–C). This showed all the ligands bound in the S_2_′, S_1_′ and S_1_ subsites, with 33RE and SG6 also extending into the S_2_ subsite. The details of the binding interactions for these ligands are described below.

### Interactions of 33RE with the N389-S_2__S′-nACE binding site

**Figure 6.**
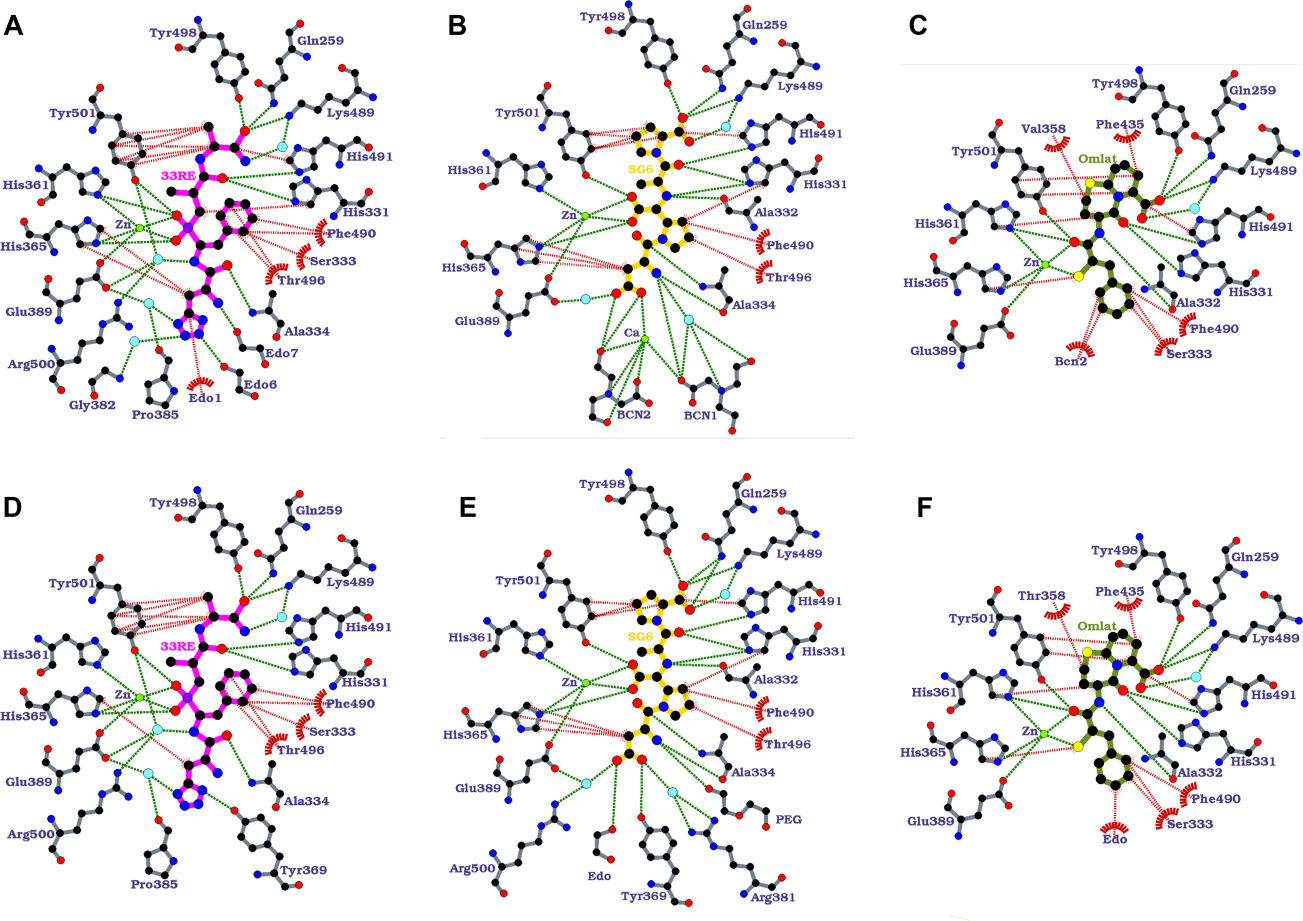
Ligplot representations of inhibitor binding interactions. Comparison of (**A**) 33RE, (**B**) SG6 and (**C**) Omapatrilat bound to N389-S_2__S′-nACE, with (**D**) 33RE (PDB 4BXK), (**E**) SG6 (PDB 6EN5) and (**F**) Omapatrilat (PDB 6H5X) wild-type N389-nACE complexes. H-bond/electrostatic and hydrophobic interactions are shown as green and red dashed lines, respectively, water molecules as cyan spheres, and red, semi-circular symbols depict residues solely involved in hydrophobic interactions.

As mentioned above, 33RE is bound in the S_2_′ to S_2_ subsites (Figure 6A) with its amide group located in the typical peptide C-terminal binding site composed of hydrogen bonding from the amide oxygen with Gln259, Lys489 and Tyr498, and a water-mediated interaction of the amide nitrogen with Lys489. The P_2_′ alanine-like residue of 33RE also forms extensive hydrophobic interactions with Tyr501 and His491.

The backbone carbonyl of the P_1_′ alanine mimic has hydrophilic interactions with the sidechains of His331 and His491, while the backbone carbon atom (that would be equivalent to a peptide backbone nitrogen) has a hydrophobic interaction with His331.

The zinc-binding group of 33RE is a phosphinic acid that replaces the P_1_ backbone carbonyl of a peptide and forms a bidentate interaction with the zinc ion as well as hydrogen bonds with Tyr501, His361 and His365. The P_1_ backbone nitrogen of 33RE interacts with a water molecule that co-ordinates with Glu389, Arg500 and Tyr501. The phenylalanine-like sidechain hydrophobically interacts with His331, Ser333, Phe490 and Thr496.

The P_2_ group of 33RE has a peptide-like backbone with the carbonyl having a hydrogen bond with Ala334. The 33RE P_2_ sidechain, a tetrazole, shows hydrophobic interactions with His365, and hydrogen bonds to two water molecules that are also bound by the Gly382 backbone nitrogen, Pro385 backbone carbonyl and Glu389 sidechain. There are also three ethylene glycol molecules (from the crystallization buffer) that interact with the 33RE P_2_ group via the backbone nitrogen, another nitrogen from the tetrazole group and a hydrophobic interaction with the tetrazole carbon atom.

### Interactions of SG6 with the N389-S_2__S′-nACE binding site

The P_2_′ and P_1_′ groups of SG6 are essentially mimics of proline and alanine, respectively. Therefore, it is not surprising that the P_2_′ carboxylic acid group has identical interactions with Gln259, Lys489, His491 and Tyr498 described above for 33RE (Figure 6B). The similarity continues where the prolyl ring has extensive hydrophobic interactions with Tyr501. The backbone carbonyl of the P_1_′ alanine residue hydrogen bonds with His331 and His491, and the nitrogen interacts with His331 and the backbone of Ala334.

The P_1_ group of SG6 differs from a peptide by having a carboxylic acid group replacing the backbone carbonyl, and this forms a bidentate interaction with the active site zinc ion, as well as having hydrogen bonds with Tyr501 and His365. The prolyl ring sidechain of SG6 has hydrophobic interactions with His331, Phe490 and Thr496.

SG6 has a P_2_ aspartate group where the backbone carbonyl and nitrogen form hydrogen bonds with the backbone nitrogen and carbonyl, respectively, of Ala334. The aspartate sidechain has hydrophobic interactions with His365, and a water-mediated hydrogen bond with Glu389. There is a complex structure (from the crystallisation buffer) of two bicine molecules, a calcium ion and water molecules that is adjacent to the SG6 P_2_ aspartate. The carboxylic acid group of the aspartate forms part of the calcium ion's coordination sphere, as well as interacting with one of the bicine molecules. In addition, the backbone nitrogen of the aspartate has direct, and water-mediated hydrogen binds with the second bicine.

### Interactions of omapatrilat with the N389-S_2__S′-nACE binding site

Omapatrilat contains atoms that mimic a peptide backbone, and this includes the P_2_′ carboxylic acid group that binds in the typical S_2_′ carboxy-terminus binding site showing direct hydrogen bonds with Gln259, Lys489 and Tyr498, a water-mediated interaction with Lys489, and hydrophobic interaction with His491 (Figure 6C). The P_2_′ ring of omapatrilat has hydrophobic interactions with Phe435 and Tyr501.

The P_1_′ ring also shows hydrophobic interactions, these are with Val358 and His361. Whereas the P_1_′ backbone carbonyl mimic hydrogen bonds to His331 and His491, and the backbone nitrogen equivalent interacts with the backbone of Ala332.

The P_1_ group of omapatrilat contains a carbonyl group that binds to the active site zinc ion as well as His361 and Tyr501. There is also a sulfur ion that binds to the zinc ion, and to His365 via a hydrophobic interaction. The P_1_ phenyl sidechain has hydrophobic interactions with Ser333, Phe490 and a bicine molecule from the crystallization buffer.

## Discussion

### Comparison of mutant structures with the wild-type

The inhibitors co-crystallised with N389-S_2__S′-nACE in this study have all been previously solved in complex with N389-nACE (33RE PDB 4BXK [18], omapatrilat PDB 6H5X [27], SG6 PDB 6EN5 and 6EN6 [25]). Therefore, comparisons can be directly made to see how the S_2__S′ mutations affect the binding interactions of these ligands.

**Figure 7.**
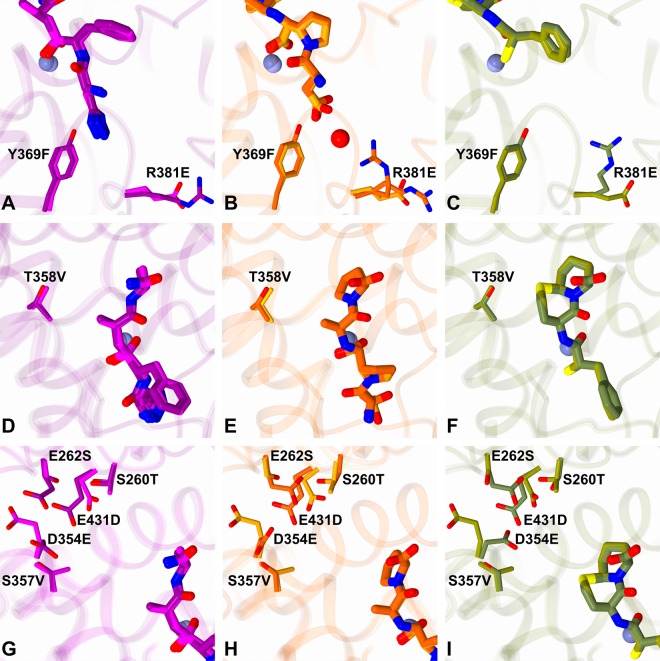
Overlay of N389-S_2__S′-nACE mutations with wild-type N389-nACE. Inhibitor complexes with N389-S_2__S′-nACE (33RE in magenta, SG6 in orange and omapatrilat in olive) and wild-type N389-nACE (33RE in dark magenta (PDB 4BXK), SG6 in orange red (PDB 6EN5) and omapatrilat in dark olive (PDB 6H5X)) are overlaid with close up views of (**A**–**C**) Non-prime, (**D**–**F**) S_1_′ and (**G**–**I**) distal S′ mutations.

N389-S_2__S′-nACE contains mutations in the S_2_ subsite (Y369F and R381E, Figure 7A–C), S_1_′ subsite (T358V, Figure 7D–F) and beyond the S_2_′ subsite (S260T, E262S, D354E, S357V and E431D, Figure 7G–I). Residue Thr358 may also have a longer-range effect on the S_2_′ subsite when there are larger P_2_′ groups such as tryptophan. As described above, these mutations have very little effect on the overall structure of the enzyme when ligands are bound. It is, therefore, not surprising that for all three inhibitors, the majority of interactions are unchanged when comparing the N389-nACE and N389-S_2__S′-nACE complexes (Figure 6). This is consistent with five of the eight mutations in N389-S_2__S′-nACE being distant from the typical S_2_′ to S_2_ subsites. In addition, an overlay of the structures show that the ligands adopt almost identical conformations in the N389-nACE and N389-S_2__S′-nACE structures (Figure 5D–F), apart from a small difference in the SG6 P_2_ aspartate sidechain as described below.

Comparison of the 33RE complex structures shows that all interactions with the P_2_′ to P_1_ groups are conserved. The only significant difference in interactions is observed with the P_2_ group of 33RE, as a result of the Y369F mutation. In the wild-type 33RE structure, the hydroxyl group of Tyr369 interacts with the tetrazole side chain of 33RE, and this interaction is obviously removed by the mutation to phenylalanine (Figures 6A, D and 7A). This is consistent with the reduction in affinity observed for S_2_-nACE compared with wild-type nACE.

The interactions with SG6 are identical in both N389-nACE and N389-S_2__S′-nACE except for those with the carboxylic acid group of the P_2_ sidechain (Figures 6B, E and 7B). In N389-nACE this group has a direct hydrogen bond with Tyr369 and a weak water-mediated interaction with Arg381 (in eight molecules of nACE from two crystal structures, the electron density for Arg381 was weak and showed a mixture of conformations, with not all able to form this interaction). These two residues are mutated to phenylalanine and glutamate, respectively, in N389-S_2__S′-nACE and are unable to interact with the carboxylic acid group. This lack of interactions from the protein is likely the cause of the change in rotation of the P_2_ group in the N389-S_2__S′-nACE structure compared with the N389-nACE complex (Figure 5E), and the subsequent binding of the calcium and bicine complex described above (Figure 6E). There is also an effect on the water molecules around the SG6 P_2_ sidechain such that in N389-S_2__S′-nACE, Arg500 is no longer involved in a water-mediated interaction. This reduction in interactions between SG6 and the protein explains the increase in *K*_i_ of SG6 for S_2_-nACE compared with nACE.

As with P_2_′ to P_1_ groups of 33RE, all the interactions with omapatrilat are conserved in both the N389-nACE and N389-S_2__S′-nACE complex structures, although one is altered (Figures 6C, F and 7F). In this case, there are no additional bonds, instead the hydrophobic interaction from the P_1_′ ring of omapatrilat with residue 358 is different because it is a threonine in N389-nACE, but mutated to valine in N389-S_2__S′-nACE. The kinetic data showed that the affinity of omapatrilat for nACE is unchanged by the mutations, which is reflected by all the interactions being conserved, and indicates that the T358V mutation essentially does not affect the strength of that hydrophobic interaction.

### The role of distal interactions in domain-selectivity

Despite the similarities between the mutant and wild-type structures, the binding affinities of 33RE, SG6, ketoACE-13 and enalaprilat were altered by mutation of the S_2__S′ residues, albeit to different degrees. This suggests that the affinities of these ligands for nACE are not only affected by direct interactions and the immediate environment of the final binding site, but also by residues more distant. In contrast, omapatrilat was unaffected by mutation and displayed sub-nanomolar inhibition for the mutants as well as nACE and cACE, which raises the question of how these distant residues can affect the binding of some ligands, but not others?

**Figure 8.**
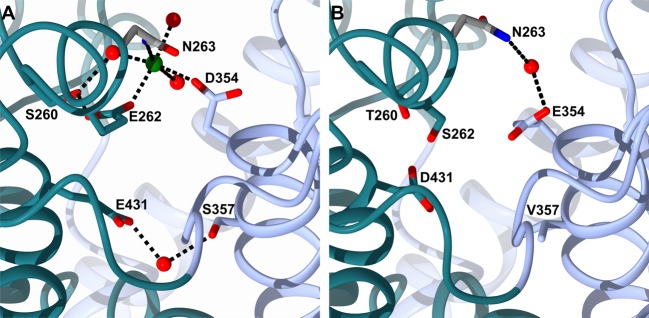
Interactions of mutated residues near the predicted hinge region. Close up view of the S′ residues included in the N389-S_2__S′-nACE mutations showing their interactions in (**A**) Wild-type N389-nACE and (**B**) N389-S_2__S′-nACE/SG6 structures. The two sub-domains are coloured light blue and dark cyan. Water molecules and magnesium ion are shown as red and green spheres, respectively.

Five of the mutated residues (Ser260, Glu262, Asp354, Ser357 and Glu431, referred to as SEDSE hereafter) are located at the edge of the S′ lobe on either side of where the two sub-domains of nACE come together (Figure 8A) and are structurally near the hinge 2 and 4 region. Since this is beyond the typical S_2_′ subsite, these residues can only have a direct involvement in binding of ligands if there is a long P_2_′ group or if the ligand extends to what would be a S_3_′ subsite. The SEDSE residues, therefore, have no ligand interactions in the structures presented here (Figure 7G–I). Examination of N389-nACE structures show that there is a water-mediated interaction between Ser357 of sub-domain 1 and Glu431 of sub-domain 2, which are two of the SEDSE residues. In some of the structures solved to date, there are direct and water-mediated interactions between Asp354 of sub-domain 1 and Glu262 of sub-domain 2. In other structures, a magnesium ion has been identified that is co-ordinated by Asp354, Asn263 (a sub-domain 2 residue conserved in cACE) and Glu262, and three water molecules, one of which is co-ordinated to Ser260. Therefore, in the presence of metal ions (in this case magnesium which probably comes from the crystallization buffer), all the SEDSE residues are involved in interactions between the two sub-domains. When these residues are mutated, as seen in Figure 8B, there is no interaction between residues 357 and 431 and, while there is still a water-mediated interaction between Asn263 and Glu354, overall the strength of the interaction between the sub-domains is weaker.

Previously, it was shown that synergism between the S_2_ subsite residues (YR), the S_1_′ T358 (which has no direct interactions) and the distal prime subsite residues (SEDSE) was responsible for the 1000-fold N-selective binding of 33RE [23], the molecular basis of which was elucidated by molecular dynamics simulations. While the interaction of Y369 with the tetrazole of 33RE was important to directly stabilize the molecule in the active site, R381 and the distal T358 and SEDSE residues were indirectly involved. R381 is located on a flexible loop, known as hinge region 3, between the two sub-domains and propelled the tipping motion of the lid region via repulsion of its R90 residue to favour cleft closure. The distal prime subsite residues increased 33RE binding affinity by providing a hydrophobic environment for the P_1_′ pseudoalanine (T358) and creating a polar sub-domain interface to drive active site closure via hinging at the neighbouring hinge regions 2 and 4. This is consistent with the sub-domain interactions described above for the crystal structures presented here.

Prior to obtaining the kinetic results presented here, it was believed that 33RE was the only ACE inhibitor to date with this mechanism of action due to some unique properties contained in its backbone. Amidation of the C-terminal pseudoalanine was thought to be crucial in conferring selectivity based on an earlier study on the parent of 33RE, RXP407 [17], whereas the weaker phosphinic acid zinc-binding group was thought to allow selection based on the inter-subsite synergism. Here, we have found, however, that SG6 (which has a completely different backbone except for the P_1_′ pseudoalanine) follows the same mechanism of action as 33RE. These two moieties are, therefore, not a requirement for N-selectivity. Although it had the same level of selectivity, SG6 was, however, much more potent than 33RE with picomolar to nanomolar affinities for all enzymes tested. This is likely due to its stronger zinc-binding (carboxylic acid) and/or C-terminal (proline with free carboxylic acid) moieties.

Previous work on ketoACE analogues where P_2_ aspartate and P_2_′ amidated pseudoalanine or -proline moieties were incorporated onto this backbone had much lower N-selectivities compared with RXP407 [24]. The kinetic results presented here confirm these findings for ketoACE-13 and suggest that it is in part caused by the absence of a P_1_′ pseudoalanine. This group is commonly found in many ACE inhibitors and is often described as being important for potency, but not necessarily domain-selectivity [44]. For more ‘traditional’ ACE inhibitors that only consist of P_1_ to P_2_′ groups, the addition of a P_1_′ pseudoalanine would likely increase binding, but not selectivity, by introducing a hydrophobic moiety in the proximity of the nACE residue T358 and its cACE counterpart V380. Although these residues are not in direct contact with the P_1_′ pseudoalanine (typically more than 4 Å away), they likely create a more favourable environment for positioning of the inhibitor and direct interaction with the zinc and conserved residues. In the case of 33RE or SG6, however, this group likely enhances the active site closure mediated by the SEDSE residues, thus increasing the selectivity relative to ketoACE-13.

**Figure 9.**
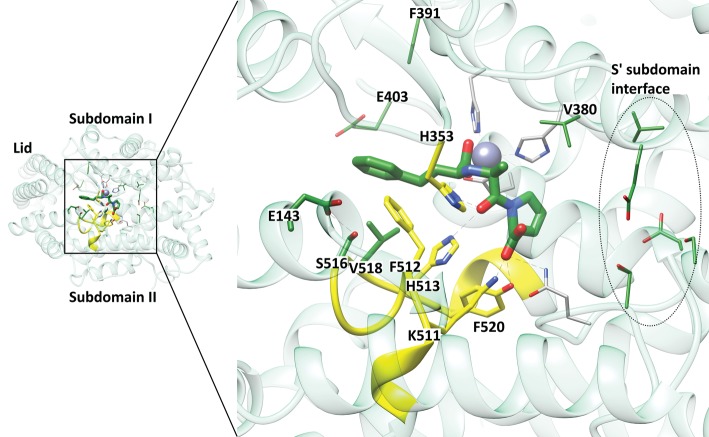
Binding of enalaprilat to cACE. Enalaprilat is shown as green sticks bound to cACE (light green ribbon, PDB ID 1UZE), the zinc ion as a grey sphere and the unique S_2_ and distal prime subsite residues as green wires. The unique cACE residues E143 and S516 interact to potentially stabilize a loop region highlighted in yellow (harbouring the unique V518) and increase the strength of enalaprilat interaction with the conserved residues (yellow sticks).

It was interesting to note that for ketoACE-13 and enalaprilat, but not omapatrilat, mutation of S_2__S′ resulted in binding affinities that were, respectively, 6-fold and 19-fold lower than that of cACE. These mutations created a binding site which was essentially equal to that of cACE and indicates that although the direct enalaprilat interactions are conserved between the two domains, the mechanism of inhibition differed. To better understand this phenomenon, the previously published structure of enalaprilat complexed to cACE (PDB ID 1UZE) [45] was carefully inspected. It is proposed that effective inhibition of nACE by enalaprilat still relies on R381 to control lid tipping and S′ to control sub-domain contact despite the absence of a P_2_ group for interaction with Y369. The P_2_ group is likely not required for potent nACE inhibition when the P_1_′ pseudoalanine and acidic P_2_′ pseudoproline moieties are present, as in enalaprilat. Since cACE is less favoured for active site closure due to repulsion at the sub-domain interface, but still effectively inhibited by enalaprilat, we hypothesized that some property distal to the active site might assist in its inhibition. The P_1_ pseudophenylalanine is present in ketoACE-13 as well as enalaprilat and interacts with nACE T496 or cACE V518. Although the more hydrophobic valine might slightly increase the strength of the P_1_ interaction, it is unlikely to explain the discrepancy between cACE and S_2__S′-nACE inhibition by enalaprilat. Interestingly, the T496/V518 residues are located on a long loop region that connects the helix harbouring nACE Y523/cACE Y501 (which binds to the inhibitor backbone) to conserved residues (nACE H491, Y498/cACE H513, Y520) which bind to the inhibitor's prime moieties (Figure 9). This loop region appears to be uniquely stabilized in the cACE enalaprilat structure by the presence of a hydrogen bond between the residing S516 (replaced by Asn in nACE) and E143 (replaced by Ser in nACE) proximal to the lid region. This interaction is only observed in some cACE crystal structures which suggests that it is transient, in line with the dynamic nature of residues around the lid region. It likely plays an indirect role during the binding process by driving the loop region's dynamics towards the bent conformation observed in the ligand-bound state so that inhibition occurs despite the barrier to sub-domain contact close to the cACE prime subsites. The two domains of ACE thus appear to have unique ways of stabilizing/guiding active site loops to favour ligand binding. Since S_2__S′-nACE results in loss of interactions important in nACE stabilization and naturally lacks those present in cACE, the affinity for enalaprilat and ketoACE-13 is lower for this mutant than either wild-type domain. It likely only offers an advantage to cACE if either the P_2_ or P_1_′ interactions are absent, as with enalaprilat and ketoACE-13, respectively, since this decreases the strength of nACE inter-subsite synergism. This effect of loop stabilization was not observed for omapatrilat which inhibited all enzymes equally, likely due to it being overruled by strong zinc chelation. Future investigations into the dynamics of these distal regions might yield insight into the divergent substrate specificities of the highly conserved ACE active sites.

## Conclusion

Typically, protein–ligand interactions are usually the focus for the design of inhibitor potency and specificity. In this study, we show that to achieve the desired domain specificity of next-generation ACE inhibitors, it is important to also consider intra-protein interactions and distal residues that the inhibitor may transiently interact with during entry into the binding site. Thus, the entire protein must be considered as a dynamic molecule and not just the active site in isolation.

While this does add to the complexity for the design process, due to the high degree of similarity between the binding sites of nACE and cACE, this new information will prove beneficial in obtaining new domain-specific inhibitors. Further study is required to fully understand these distal effects and this will help with inhibitor development.

## Database Depositions

The atomic co-ordinates and structure factors have been deposited in the RCSB Protein Data Bank (www.rcsb.org/pdb) with accession numbers 6TT1, 6TT3 and 6TT4. The atomic co-ordinates and experimental data will be released upon article publication.

## Abbreviations

Abz: o-aminobenzoyl
ACE: angiotensin-1 converting enzyme
AcSDKP: acetyl-Ser-Asp-Lys-Pro
Aβ: amyloid beta-peptide
CHO: Chinese hamster ovary cells
DMSO: dimethyl sulfoxide
Dnp: dinitrophenyl
GnRH: gonadotropin-releasing hormone
LH-RH: luteinizing hormone-releasing hormone
PDB: Protein Data Bank
RAAS: renin-angiotensin-aldosterone system
